# Strongly lensed repeating fast radio bursts as precision probes of the universe

**DOI:** 10.1038/s41467-018-06303-0

**Published:** 2018-09-20

**Authors:** Zheng-Xiang Li, He Gao, Xu-Heng Ding, Guo-Jian Wang, Bing Zhang

**Affiliations:** 1 Department of Astronomy, Beijing Normal University, 100875 Beijing, China; 2 School of Physics and Technology, Wuhan University, 430072 Wuhan, China; 3 Department of Physics and Astronomy, University of Nevada, Las Vegas, NV 89154 USA; 4 National Astronomical Observatories of China, Chinese Academy of Sciences, 100012 Beijing, China; 5 Department of Astronomy, School of Physics and Kavli Institute for Astronomy and Astrophysics, Peking University, 100871 Beijing, China

## Abstract

Fast radio bursts (FRBs), bright transients with millisecond durations at ∼GHz and typical redshifts probably >0.8, are likely to be gravitationally lensed by intervening galaxies. Since the time delay between images of strongly lensed FRB can be measured to extremely high precision because of the large ratio ∼10^9^ between the typical galaxy-lensing delay time $\sim{\cal O}$ (10 days) and the width of bursts $\sim{\cal O}$ (ms), we propose strongly lensed FRBs as precision probes of the universe. We show that, within the flat ΛCDM model, the Hubble constant *H*_0_ can be constrained with a ~0.91% uncertainty from 10 such systems probably observed with the square kilometer array (SKA) in <30 years. More importantly, the cosmic curvature can be model independently constrained to a precision of ∼0.076. This constraint can directly test the validity of the cosmological principle and break the intractable degeneracy between the cosmic curvature and dark energy.

## Abstract (web summary)

Fast radio bursts (FRBs) are likely to be gravitationally lensed by intervening galaxies. Here, the authors propose to make accurate measurements of time delays between images of lensed FRBs as a powerful probe for precision cosmology.

## Introduction

Fast radio bursts (FRBs) are bright transients with millisecond durations at ~GHz frequencies, whose physical origin is subject to intense debate^1,2^. Most FRBs are located at high galactic latitudes and have anomalously large dispersion measures (DMs). Attributing DM to an intergalactic medium origin, the corresponding redshifts *z* are typically >0.8. Up to now, more than 30 FRBs have been published^3^. One of them, FRB 121102, shows a repeating feature^4^. The repetition of FRB 121102 enables high-time-resolution radio interferometric observations to directly image the bursts, leading to the localization of the source in a star-forming galaxy at *z* = 0.19273 with sub-arcsecond accuracy^5^. The cosmological origin of the repeating FRBs is thus confirmed. For other FRBs, although no well-established evidence has been published, they are also strongly suggested to be of a cosmological origin, due to their all-sky distribution and their anomalously large values of DMs^2^. It is possible that all FRBs might be repeating, and only the brightest ones are observable. On the other hand, it is also possible that repeating and non-repeating FRBs may originate from different progenitors^6^. Interestingly, these transient radio sources are likely lensed from small to large scales, for example, through plasma lensing in their host galaxies^7^, gravitational microlensing by an isolated and extragalactic stellar-mass compact object^8,9^, and strong gravitational lensing by an intervening galaxy^10,11^. Here we only focus on the possibility of strongly gravitationally lensed FRBs and their applications to conduct cosmography. Therefore, in our following analysis, “lensed FRBs” only refers to the case that an FRB is strongly gravitationally lensed by an intervening galaxy. For a lens galaxy with the mass of dark matter halo $\sim10^{12}M_ \odot h^{ - 1}$ (*h* is the Hubble constant in units of 100 km s^−1^ Mpc^−1^), the typical time delay and angular separation between different images of lensed FRBs are $\sim{\cal O}$ (10 days) and of the order arcseconds, respectively. These multiple images of lensed FRBs cannot be resolved by radio survey telescopes since their typical angular resolution is of the order of 10 arcmin. Therefore, a lensed non-repeating FRB source may be observed as a repeating FRB source, showing two to four bursts with respective time delays of several days. The DM value and the scatter broadening of each burst could be slightly or even significantly different from each other depending on the plasma properties along different lines of sight (LOS). It is therefore difficult to identify the lensed non-repeating FRBs. However, if a repeating FRB is strongly lensed by an intervening galaxy, a series of image multiplets from the same source will exhibit a fixed pattern in their mutual time delays, appearing over and over again as we detect the repeating bursts^11^. Observations of FRB 121102 in radio and its counterpart in optical indicate that this repeater is not lensed (no intervening lens galaxy or multiple images of the host are observed) and the intrinsic repetition happens randomly. Therefore, a fixed temporal pattern associated with a future repeating FRB source would be a smoking-gun signature that it is strongly lensed. Each burst emitted from the source would travel through different paths to reach to the observer with time delays. If these lensed bursts can be imaged, they should appear as different images in the sky. Their spectra and lightcurves might be slightly different from each other because of different paths they traveled through, so that the morphology of bursts may not be the main feature to identify lensed FRBs. For a series of randomly generated repeating bursts, the intrinsic time difference between two adjacent bursts should be the same for all lensed (two or four) images. Therefore, a fixed time pattern of all the repeating bursts is the most robust evidence for identifying a lensed FRB system. Once a survey telescope registered a fixed time pattern repeating two or four times with a delay $\sim{\cal O}$ (10 days), one could then employ more powerful radio telescopes such as very large array (VLA) or the future SKA to observe more repetitions and resolve multiple images of the bursts. Meanwhile, one could observe the source using optical and near-infrared (IR) telescopes to identify an intervening lens galaxy near the LOS as well as the multiple images of the host galaxy (Einstein ring or arcs) with angular separations of the order of arcseconds. If the image locations in both radio and optical (or near-IR) bands match each other, in combination with the fixed time-delay repetition pattern mentioned above, a lensed FRB system can be confirmed and the host and the lens galaxies identified.

Current FRB observations suggest a sufficiently high all-sky FRB rate of ~10^3^–10^4^ per day^2,12^. Upcoming surveys such as the Swinburne University of Technology’s digital backend for the Molonglo Observatory Synthesis Telescope array (UTMOST)^13^, the Hydrogen Intensity and Real-time Analysis eXperiment (HIRAX)^14^, the Canadian Hydrogen Intensity Mapping Experiment (CHIME)^15^, and especially the SKA project^16^ will map a considerable fraction of the sky with a detection rate of FRBs of >100 per day^17^. For an FRB happening at *z* > 1, the probability for it to be strongly lensed is ~a few ×10^−4 ^^18^. As a result, future radio surveys, such as SKA, will have the ability to discover >10 strongly lensed FRBs per year^9–11^. According to the current data, at least 3% (1/30) observed FRBs are repeating FRBs. With a conservative estimate, ~10 strongly lensed repeating FRBs are expected to be accumulated within <30 years with the operation of SKA.

Owing to the small ratio (~10^−9^) between the short duration of each burst $\sim{\cal O}$ (ms) and the typical galaxy-lensing delay time $\sim{\cal O}$ (10 days), time delays between images of these systems can be measured to great precision. Moreover, due to overwhelmingly accurate localizations of lensed FRB images from deep VLA observations (or future SKA observations) and clean high-resolution images of the host galaxy without a dazzling active galactic nucleus (AGN), the mass profile of the lens can be also modeled with high precision. Therefore, we propose that lensed FRB systems can be a powerful probe for studying cosmology. Lensing theory predicts that the difference in arrival time between image A and image B, that is, the “time delay” Δ*τ*_AB_, is expressed as

1 $${\mathrm{\Delta }}\tau _{{\mathrm{AB}}} = \frac{{{\mathrm{\Delta \Phi }}_{{\mathrm{AB}}}}}{c}D_{{\mathrm{\Delta }}t} = \frac{{{\mathrm{\Delta \Phi }}_{{\mathrm{AB}}}}}{c} \cdot (1 + z_{\mathrm{l}})\frac{{D_{\mathrm{l}}^{\mathrm{A}}D_{\mathrm{s}}^{\mathrm{A}}}}{{D_{\mathrm{ls}}^{\mathrm{A}}}},$$

where ΔΦ_AB_ is the Fermat potential difference between the two image positions, *c* is the speed of light, *D*_Δ*t*_ the so-called “time-delay distance” is just a multiplicative combination of the three angular diameter distances ($D_{\mathrm{l}}^{\mathrm{A}}$: from the observer to the lens, $D_{\mathrm{s}}^{\mathrm{A}}$: from the observer to the source, $D_{\mathrm{ls}}^{\mathrm{A}}$: from the lens to the source), and *z*_l_ is the redshift of lens. This quantity has the dimension of distance and is inversely proportional to the Hubble constant, *H*_0_, which sets the age and length scale for the present universe and is one of the most important parameters for cosmology. Therefore, the time-delay distance *D*_Δ*t*_ is primarily sensitive to *H*_0_ and that measured from lensed quasar systems has been used to measure the Hubble constant. Moreover, the relations among these three angular diameter distances are highly dependent on the geometric properties of the space. We introduce dimensionless comoving angular diameter distances, $d_{\mathrm{l}} \equiv d(0,z_{\mathrm{l}}) \equiv (1 + z_{\mathrm{l}})H_0D_{\mathrm{l}}^{\mathrm{A}}/c$, $d_{\mathrm{s}} \equiv d(0,z_{\mathrm{s}}) \equiv (1 + z_{\mathrm{s}})H_0D_{\mathrm{s}}^{\mathrm{A}}/c$, and $d_{\mathrm{ls}} \equiv d(z_{\mathrm{l}},z_{\mathrm{s}}) \equiv (1 + z_{\mathrm{s}})H_0D_{\mathrm{ls}}^{\mathrm{A}}/c$ (*z*_s_ is the redshift of source), to illustrate this. For example, qualitatively and intuitively, *d*_s_ is greater, equal to, or smaller than *d*_l_ + *d*_ls_ if the space is open, flat, or closed, respectively (see Methods section for quantitative details). Therefore, in combination with distances from type Ia supernova (SNe Ia) observations, the time-delay distance can be used to directly measure the spatial curvature Ω_*k*_ in a cosmological model-independent manner. Decades of observations have ushered in the era of precision cosmology. The flat Λ CDM model is found to be consistent with essentially all the conservational constraints. Yet, recent direct local distance ladder measurements of *H*_0_ have reached a 2.4% precise measurement: *H*_0_ = 73.24 ± 1.74 km s^−1^ Mpc^−1 ^^19^, which greatly increased the tension with respect to the latest Planck-inferred value (*H*_0_ = 67.27 ± 0.66 km s^−1^ Mpc^−1^)^20^ to 3.4*σ*. Lensed FRBs, as a powerful probe and completely independent dataset based on a different physical phenomenon, would provide complementary information and therefore are of vital importance to clarify this issue.

## Results

**Fig. 1:**
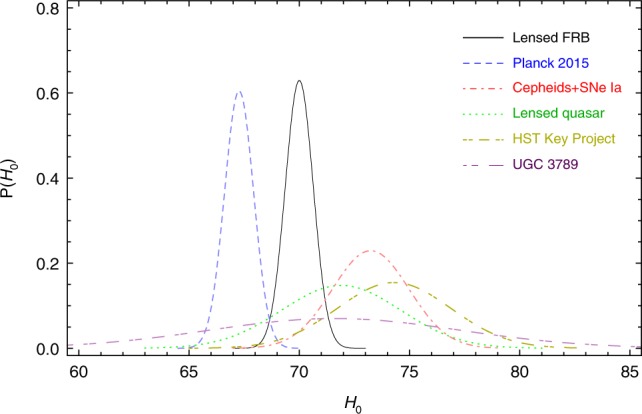
Probability distribution functions (PDFs) of the Hubble constant constrained from 10 lensed FRBs and some other currently available observations. Besides the result obtained from 10 lensed FRB systems in this work (the black solid line), from top to bottom the lines represent *H*_0_ inferred from the Planck satellite CMB measurements (67.27 ± 0.66 km s^−1^ Mpc^−1^) ^20^, local distance measurements (73.24 ± 1.74 km s^−1^ Mpc^−1^)^19^, time-delay cosmography of strongly lensed quasars $\left( {71.9_{ - 3.0}^{ + 2.4}\,{\mathrm{km}}\,{\mathrm{s}}^{ - {\mathrm{1}}}\,{\mathrm{Mpc}}^{ - {\mathrm{1}}}} \right)$^21^, distance measurements from the Hubble Space Telescope (HST) key project (74.3 ± 2.6 km s^−1^ Mpc^−1^)^55^, and VLBI observations of water masers orbiting within the accretion disc of UCG 3789 (71.6 ± 5.7 km s^−1^ Mpc^−1^)^56^, respectively

In order to investigate the constraining power of lensed FRBs on some fundamental cosmological parameters, we perform a series of simulations with the proper inputs in the following three aspects (see Methods section for details): (i) the redshift distribution of incoming FRBs; (ii) for a source at redshift *z*_s_, the lens redshift *z*_l_ to produce the maximal differential lensing probability; (iii) the uncertainty of each factor contributing to the accuracy of time-delay distance measurement. Since the time-delay distance is very sensitive to the Hubble constant, we estimate the constraining power on *H*_0_ by simulating 10 lensed FRBs in the flat ΛCDM with the matter density being fixed as Ω_m_ = 0.3. With the assumed fiducial model (flat ΛCDM model with *H*_0_ = 70 km s^−1^ Mpc^−1^ and Ω_m_ = 0.3) and three factors outlined above, we performed 10,000 simulations, each containing 10 lensed FRB systems and obtained the probability distribution of the estimated *H*_0_. Two different redshift distributions, *N*_const_(*z*) and *N*_SFH_(*z*), are considered (see Methods section for details). They do not lead to significant differences in the constraint on *H*_0_ and consistently give stringent constraints with a ~0.91% uncertainty. Results are shown in Fig. 1. It is suggested that compared to the currently available results, ~10 lensed FRBs will have obvious predominance in precision in constraining *H*_0_. For instance, it improves by a factor ~5 with respect to the current state-of-the-art case of lensed quasars^21^.

**Fig. 2:**
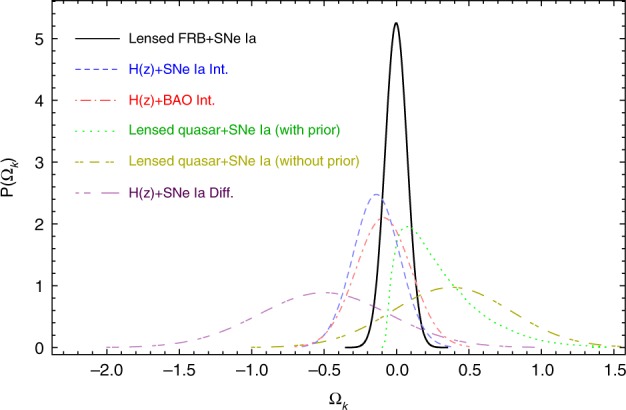
Model-independent probability distribution functions (PDFs) of the cosmic curvature estimated from 10 lensed FRBs and some other currently available observations. Besides the result obtained from 10 lensed FRB systems in this work (the black solid line), from top to bottom the lines are Ω_*k*_ inferred from the integral method with expansion rate (i.e., the Hubble parameter *H*(*z*)) and SNe Ia observations (−0.140 ± 0.161)^57^, the integral method with expansion rate and BAO observations (−0.09 ± 0.19)^58^, distance sum rule with the prior $\Omega _k > - 0.1\left( {0.25_{ - 0.33}^{ + 0.72}} \right)$^25^, distance sum rule without the prior $\Omega _k > - 0.1\left( { - 0.38_{ - 0.84}^{ + 1.01}} \right)$^25^, and the differential approach with the expansion rate and SNe Ia observations $\left( { - 0.50_{ - 0.36}^{ + 0.54}} \right)$^59^, respectively

In addition to constraining the Hubble constant within the flat ΛCDM model, one can also give a model-independent estimate of the cosmic curvature using lensed FRBs. As one of the most important parameters in cosmology, a precise estimation of the spatial curvature is essential for justifying whether the Friedmann–Lemaître–Robertson–Walker (FLRW) metric could exactly characterize the background of the universe^22^, and for studying some fundamental issues such as cosmic evolution and dark energy property^23^. In the FLRW metric, it was found that distances along null geodesics satisfy a specific sum rule^24^, which has been proposed to test the validity of the homogeneous and isotropic background^25^. More recently, with an upgraded distance sum rule, time-delay distance measurements from lensed quasars were proposed to test the FLRW metric and estimate the cosmic curvature (see Methods section for details)^26^. Here in combination with ~4000 SNe Ia observations from the near-future dark energy survey, we examined the ability of the lensed FRBs for constraining the cosmic curvature. We found that the constraints from lensed FRBs with the two considered redshift distributions are very similar and the spatial curvature parameter can be constrained to a precision of ~0.076. Results from lensed FRBs and other currently available model-independent methods are presented in Fig. 2. Again, in this model-independent domain, lensed FRBs are the most promising tools for constraining the cosmic curvature. Moreover, the precision of the results from lensed FRBs potentially approaches that inferred from the Planck satellite observations within the standard ΛCDM model, where Ω_*k*_ = −0.004 ± 0.015 was obtained^20^.

## Discussion

Here we propose strongly lensed repeating FRBs as a precision cosmological probe. Representatively, we investigate the constraining power of lensed FRB systems observed in the near future on two of the most important cosmological parameters, Hubble constant *H*_0_ and cosmic curvature Ω_*k*_. For *H*_0_, we obtained that it can be constrained with a relative ~0.91% uncertainty from 10 lensed FRB systems. This promising constraint with sub-percent uncertainty level suggests that lensed FRBs, as a powerful probe and completely independent dataset based on a different physical phenomenon, would provide complementary information and therefore are of vital importance to clarify the tension between the latest Planck-inferred *H*_0_ and the one from direct local distance ladder observations. For Ω_*k*_, it can be constrained to a precision of ~0.076 from 10 lensed FRB systems in a model-independent way on the basis of the distance sum rule. This result is the most precise one in the field of model-independent estimations for the cosmic curvature and, on the one hand, will provide a stringent direct test for the validity of the FLRW metric. On the other hand, this constraint with considerable precision is helpful to break the degeneracy between the cosmic curvature and dark energy, and thus is conducive for investigating the nature of dark sectors of the universe. Moreover, having model-dependent and direct measurements of the same quantity is of utmost importance. In the absence of significant systematic errors, if the standard cosmological model is the correct one, indirect (model-dependent) and direct (model-independent) constraints on this parameter should be consistent. If they were significantly inconsistent, this would provide evidence of physics beyond the standard model or unaccounted systematic errors. Strongly lensed FRBs can help to reach such a goal.

## Methods

In order to examine the potential of using lensed FRBs as cosmological probes, three related aspects need to be addressed: (i) the redshift distribution of the incoming FRBs; (ii) for a source at redshift *z*_s_, the lens redshift *z*_l_ to produce the maximal differential lensing probability; (iii) the uncertainties of different factors contributing to the accuracy of time-delay distance measurements. We discuss these three items one by one. In addition, we also introduce the distance sum rule for estimating the cosmic curvature.

### FRB redshift distribution

We consider two possible scenarios suggested in ref ^9^. The first one invokes a constant comoving number density, so that the number of FRBs in a shell of width d*z* at redshift *z* is proportional to the comoving volume of the shell *dV*(*z*)^27^. By introducing a Gaussian cutoff at some redshift *z*_cut_ to represent an instrumental signal-to-noise threshold, the constant-density distribution function *N*_const_(*z*) is expressed as

2 $$N_{{\mathrm{const}}}(z) = {\cal N}_{{\mathrm{const}}}\frac{{\chi ^2(z)}}{{H(z)(1 + z)}}e^{ - D^{{\mathrm{L}}^{\mathrm{2}}}(z)/\left[ {2D^{{\mathrm{L}}^{\mathrm{2}}}(z_{{\mathrm{cut}}})} \right]},$$

where *χ*(*z*) is the comoving distance and *D*^L^ is the luminosity distance. ${\cal N}_{{\mathrm{const}}}$ is a normalization factor to ensure that the integration of *N*_const_(*z*) is unity and *H*(*z*) is the Hubble parameter at redshift *z*. The second distribution requires that FRBs follow the star-formation history (SFH)^13^, so that

3 $$N_{{\mathrm{SFH}}}(z) = {\cal N}_{{\mathrm{SFH}}}\frac{{\dot \rho _ \ast (z)\chi ^2(z)}}{{H(z)(1 + z)}}e^{ - D^{{\mathrm{L}}^{\mathrm{2}}}(z)/\left[ {2D^{{\mathrm{L}}^{\mathrm{2}}}(z_{{\mathrm{cut}}})} \right]},$$

where ${\cal N}_{{\mathrm{SFH}}}$ is the normalization factor and is chosen to have *N*_SFH_(*z*) integrated to unity. The density of SFH is parametrized as

4 $$\dot \rho _ \ast (z) = h\frac{{\alpha + \beta z}}{{1 + (z/\gamma )^\delta }},$$

with *α* = 0.017, *β* = 0.13, *γ* = 3.3, *δ* = 5.3, and *h* = 0.7^28,29^. For redshifts of currently available FRBs, different from previous estimation using a simple relataion between DM and *z* proposed by Ioka^30^, we re-estimate them with a more precise DM–*z* relation given in ref ^31^. It is found that the inferred *z* values are systematically greater than previously estimated ones, which are typically >0.8 and with several FRBs having *z* > 1 even after properly subtracting the DM contribution from the FRB host. In this case, for these two FRB distribution functions, a cutoff *z*_cut_ = 1 is chosen to match redshifts of currently detected events. In our analysis, *N*_const_ and *N*_SFH_ are employed to investigate whether cosmological implications from lensed FRBs are dependent on the assumed redshift distributions.

### Lensing probability

According to the lensing theory^32^, the probability for a distant source at redshift *z*_s_ lensed by an intervening dark matter halo is

5 $$P = {\int}_0^{z_{\mathrm{s}}} dz_{\mathrm{l}}\frac{{dD^{\mathrm{p}}}}{{dz_{\mathrm{l}}}}{\int}_0^\infty \sigma (M,z_{\mathrm{l}})n(M,z_{\mathrm{l}})dM,$$

where *dD*^p^/*dz*_l_ is the proper distance interval, *σ*(*M*, *z*_l_) is the lensing cross-section of a dark matter halo with its mass and redshift being *M* and *z*_l_, respectively, *n*(*M*, *z*_l_)*dM* is the proper number density of the deflectors with masses between *M* and *M* + *dM*. For a singular isothermal sphere (SIS) lens, the cross-section producing two images with a flux ratio being smaller than a given threshold *r* is^33^

6 $$\sigma ( < r) = 16\pi ^3\left( {\frac{{\sigma _{\mathrm{v}}}}{c}} \right)^4\left( {\frac{{r - 1}}{{r + 1}}} \right)^2\left( {\frac{{D_{\mathrm{l}}^{\mathrm{A}}D_{\mathrm{ls}}^{\mathrm{A}}}}{{D_{\mathrm{s}}^{\mathrm{A}}}}} \right)^2,$$

where *σ*_v_ is the velocity dispersion. Moreover, the comoving number density of dark matter halos within the mass range (*M*, *M* + *dM*) at redshift *z* is

7 $$n(M,z)dM = \frac{{\rho _0}}{M}f(M,z)dM,$$

where *ρ*_0_ is the present value of the mean mass density in the universe, and *f*(*M*, *z*)*dM* is the Press–Schechter function^34^. For any FRB at redshift *z*_s_ following the distribution *N*_const_ or *N*_SFH_, we determine the lens redshift *z*_l_ by maximizing the differential lensing probability, *dP*/*dz*. Assuming an SIS-like lens halo of mass $M = 10^{12}M_ \odot h^{ - 1}$ (*h* = *H*_0_/100 km s^−1^ Mpc^−1^) and *r* ≤ 5, the function of the lens redshift *z*_*l*_ producing the maximal differential lensing probability with respect to the source reshift *z*_s_ was shown in Fig. 2 of ref ^10^. In our analysis, this function is used to determine the lens redshift *z*_l_ for any given source at redshift *z*_s_.

### Uncertainty contribution

In order to estimate the time-delay distance from individual lensing systems for an accurate cosmography, as suggested in Eq. (1), it has been recognized that three key analysis steps should be carried out^35,36^: that is, (1) time-delay measurement, (2) lens galaxy mass modeling, which can be used to predict the Fermat potential differences, and (3) the line of sight (LOS) environment modeling, which is adopted to account for the weak lensing effects due to massive structures in the lens plane and along the LOS.

The differences in the arrival time between images can be precisely measured for a lensed FRB system since the short duration of each burst $\sim{\cal O}$ (ms) is much smaller (~10^−9^) than the typical galaxy-lensing time delay $\sim{\cal O}$ (10 days). Therefore, errors of time-delay measurements for lensed FRBs are negligible. Compared to the best 3% uncertainty of time-delay measurements in traditional lensed quasars^21,37^, the precision for the case of FRBs is greatly improved.

For the lens galaxy mass modeling, it requires a high-resolution, good-quality image of the lensed host galaxy and accurate localizations of the lensed FRB images. The advantage of a lensed FRB system is that it does not have a bright AGN, so that clean host images can be obtained before or after FRB. In practice, once a strongly lensed repeating FRB is identified by a large field-of-view radio survey program (e.g., CHIME), images of the lensed FRBs can be accurately localized from the deep follow-up observations with VLBI or SKA. High-quality optical images of the host galaxy can be obtained from follow-up facilities such as HST or the near-future James Webb Space Telescope (JWST), which can be used to study the mass distribution of the deflector with lens modeling techniques.

**Fig. 3:**
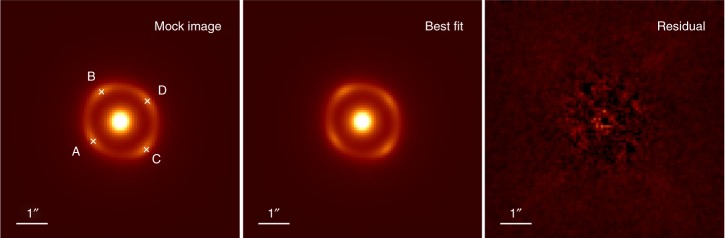
Left: Simulation results based on HST, WFC3/F160w with image drizzled to 0.08′′. Middle: Best-fit image. Right: Residual map

**Fig. 4:**
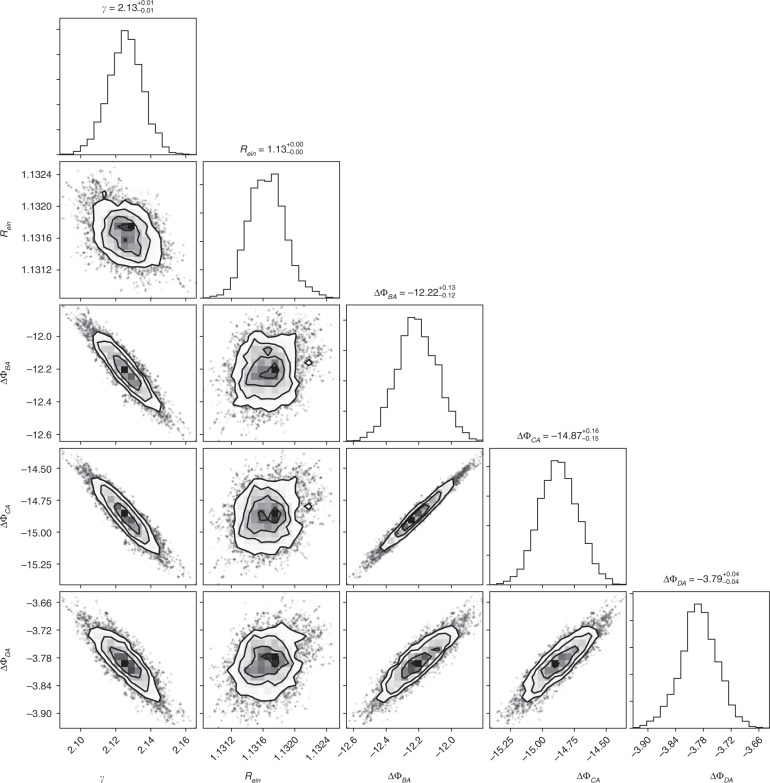
The contours of parameters inferred from the MCMC technique. The demonstrated parameters are the Einstein radius *R*_ein_ (in units of arcsecond), power-law mass profile slope *γ*, and differences of Fermat potentials between each pair of images. Note that Fermat potentials have no units, and we have re-scaled their values to better present the uncertainty level

**Fig. 5:**
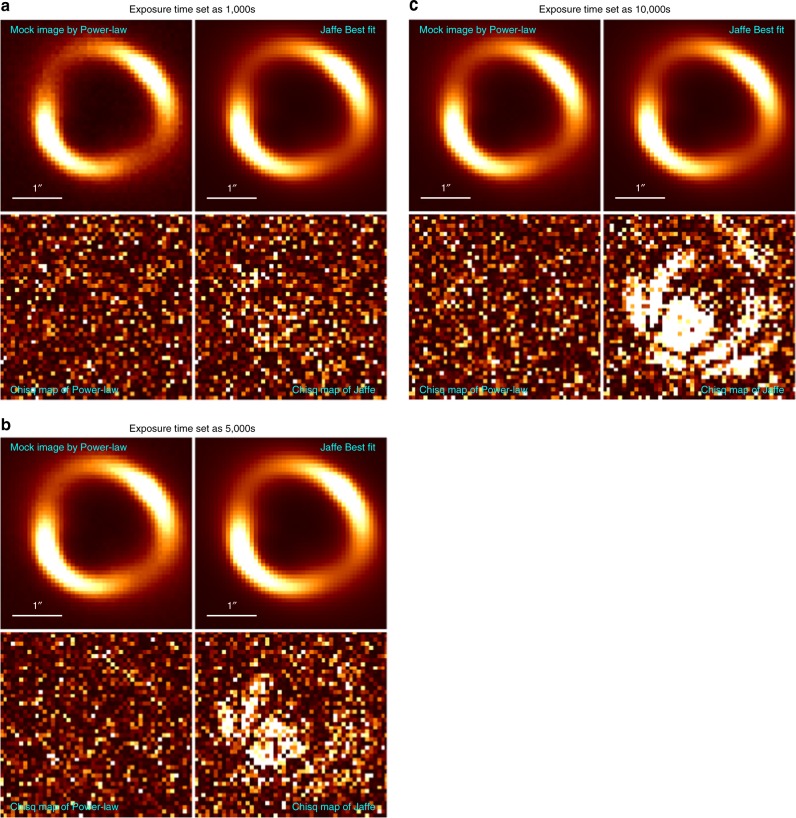
Results of the simulation tests by generating the lensed arc with a power-law mass distribution model but fitting with the Jaffe model

**Fig. 6:**
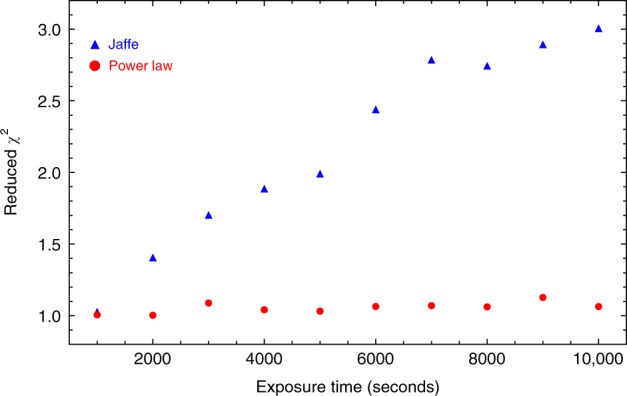
The reduced *χ*^2^ values by fitting the mock data with true model (i.e., power-law) and the wrong model (i.e., Jaffe) when varying the exposure time from 1000 to 10,000 s

In order to quantitatively estimate the uncertainty level of lens modeling from integrated lensed host image without a dazzling AGN, we carry out a series of simulations. First, we generate mock-lensed images following the industrial standard as introduced in refs ^21,38^. Specifically, in our simulation, the Sérsic profile^39^ is used to describe light profiles of the source (background) and the lens (foreground) galaxies. For lens mass profile, it is assumed to follow the power-law mass distribution of elliptical galaxies. Images are supposed to be observed by HST using the Wide Field Camera 3 (WFC3) IR channel in the F160W band. The settings related to the quality of mock images, such as the exposure time and drizzling process, are chosen based on the H0LiCOW program. Even though, in the simulation, FRB is non-luminous in optical/IR band and thus does not contribute any light to the surface brightness of images, locations of FRB images are considered to calculate the difference of the Fermat potential between each point source in the image plane. The final simulated image is shown in Fig. 3 (left panel). We apply a Monte Carlo Markov Chain (MCMC) approach to find the best-fit parameters (and parameter uncertainty) for the light and mass profiles of the source and lens galaxies, by fitting the mock image with Glafic^40^. The best-fit and the residual maps are shown in the middle and the right panel of Fig. 3, respectively. We then calculate the differences of Fermat potentials between each pair of images based on the fitting results and plot their contours together with the slope of the power-law lens mass profile and the Einstein radius *R*_ein_ (see Fig. 4). There appears to be an obvious degeneracy between the slope of the power-law mass profile *γ* and the Fermat potential differences (ΔΦ_BA_, ΔΦ_CA_, and ΔΦ_DA_), which is understandable since the latter are derived from the fitting results and theoretically the mass slope *γ* determines the lens mass distribution and thus determines the Fermat potential distribution (see Eq. (38) in ref. ^41^). Additional observational information, such as stellar velocity dispersion of the lens galaxy, can be possibly collected for providing complementary constraints on *γ* and thus are helpful for reducing uncertainties of Fermat potential differences. More importantly, as suggested in Fig. 4, the uncertainty of Fermat potential difference between two point sources is about 1%. In time-delay cosmography, the concerned parameters, such as *H*_0_ and Ω_*k*_, are inferred based on the combination of time-delay and Fermat potential difference between each pair of images. For a quadruply lensed system, we found that the uncertainty from lens modeling on cosmological parameters (*H*_0_ and Ω_*k*_) inference is 0.8%. Here we choose a power-law model to fit the lens mass profile. In the literature, it has been noted that adopting the power-law mass distribution as a specific prior might lead to a potential bias due to mass-sheet degeneracy^42–44^. However, it also has been argued that such a bias could be reduced by carefully taking into account kinematic constraints and additional sources of systematic uncertainty^44^. We want to point out here that this 0.8% uncertainty level is valid when fitting the lens with a correct parameterized model (i.e., we generate the mock image with power-law lens mass profile and fit the image also with a power-law model). Incorrectness of the lens model would lead to potential bias in the inference of *H*_0_, where greater deviation of the models leading to more significant bias. For instance, when we use the “Jaffe” model^45,46^ to fit the mock lensing system shown in the left panel of Fig. 3, where a power-law profile is considered, we even find 6–10% bias in the inference of *H*_0_. In practice, fortunately, high-quality optical/IR image of the source-lens system could help us to avoid choosing obviously wrong models. To briefly demonstrate this, we use the power-law profile to simulate the lens arc but use the “Jaffe” mass model (i.e., a wrong model) to fit the mock arc. We find that when the exposure time is longer than 5000 s, the *χ*^2^ map for the “Jaffe” model starts to be prominent (see Fig. 5) and the reduced *χ*^2^ values are much larger than the power-law ones (see Fig. 6). This simple test demonstrates that the performance of different lens models could be distinguished when the quality of observed images is high enough (e.g., the exposure time is longer than 5000 s with the HST). For current available systems studied by the H0LiCOW program, the typical exposure time with the HST is ~10^4^ s^21^. It is reasonable to expect that for each interesting lensed FRB system, extremely high-quality images can be obtained from the HST or the near-future JWST to distinguish among different lens models. Moreover, to mitigate such a potential bias, different parameterized models are often adopted so that a joint-consistent inference could be achieved. For example, in H0LiCOW IV^47^, besides the power-law model, some other popular mass models were also adopted and resulted in consistent inferences with the power-law ones. Overall, we conclude that for the near-future lensed FRB systems of great interest, lens mass modeling would contribute an uncertainty at a 0.8% level.

The last ingredient of uncertainty contribution for time-delay cosmography is the one from LOS environment modeling. In this issue, a troublesome point is that the external convergence (*κ*_ext_), resulting from the mass distributed outside of the lens galaxy but close in projection to the lens system along the LOS, does not change observable quantities of a lens system (i.e., image positions, flux ratios for point sources, and the image shapes for extended sources) but affects the predicted time delays by a factor of (1 − *κ*_ext_) when we fit the lens and source models to observations. Consequently, the true *D*_Δ*t*_ is related to the modeled one via $D_{{\mathrm{\Delta }}t} = D_{{\mathrm{\Delta }}t}^{{\mathrm{model}}}/(1 - \kappa _{{\mathrm{ext}}})$. For the lens HE 0435-1223^48^, by using various combinations of relevant informative weighing schemes for the galaxy counts^49^ and ray tracing through the Millennium Simulation^50^, it was found that the most robust estimate of *κ*_ext_ has a median value $\kappa _{{\mathrm{ext}}}^{{\mathrm{med}}} = 0.004$ and a standard deviation *σ*_*κ*_ = 0.025, which corresponds to a 2.5% uncertainty on the time-delay distance^51^. More recently, using deep *r*-band images from Subaru-Suprime-Cam and an inpainting technique and Multi-Scale Entropy filtering algorithm, a weak gravitational lensing measurement of the external convergence along the LOS to HE 0435-1223 was achieved $\kappa _{{\mathrm{ext}}} = - 0.012_{ - 0.013}^{ + 0.020}$, which corresponds to ~1.6% uncertainty on the time-delay distance^52^. Furthermore, the distribution of *κ*_ext_ is robust to choices of weights, apertures, and flux limits, up to an impact of 0.5% on the inferred time-delay distance^53^. Here we assume that for lensed FRB systems, the LOS environment contributes a systematic uncertainty at an averaged 2% level to the inferred time-delay distance, expecting that every lensed FRB system is given enough attention by the community so that we can combine auxiliary follow-up data from facilities at different wavelengths and other available simulations with convergence maps. It is still worth noting that larger LOS systematic uncertainty could lead to larger error bars of cosmological parameters. In addition to the distribution of mass external to the lens, the mass distribution in the outskirt of the lens halo, in which there are little optical light traces, might lead to errors in the inferred time-delay distance at the percent level and it was shown that weak gravitational lensing and simulations may help to reduce these uncertainties^54^.

### Distance sum rule

According to the cosmological principle, the space of the universe is homogeneous and isotropic at large scales. In this case, the spacetime can be described by the following FLRW metric:

8 $$ds^2 = - c^2{d}t^2 + a^2(t)\left( {\frac{{{d}r^2}}{{1 - Kr^2}} + r^2{d}{\mathrm{\Omega }}^2} \right),$$

where *a*(*t*) is the scale factor and *K* is a constant characterizing the geometry of three-dimensional space. In this metric, the dimensionless comoving angular diameter distance of a source at redshift *z*_s_ as observed at redshift *z*_l_ is written as

9 $$d(z_{\mathrm{l}},z_{\mathrm{s}}) = \frac{1}{{\sqrt {\left| {{\mathrm{\Omega }}_k} \right|} }}S_K\left( {\sqrt {\left| {{\mathrm{\Omega }}_k} \right|} {\int}_{z_{\mathrm{l}}}^{z_{\mathrm{s}}} \frac{{dx}}{{E(x)}}} \right),$$

where ${\mathrm{\Omega }}_k \equiv - K/H_0^2a_0^2$ (*a*_0_ = *a*(0) is the present value of the scale factor),

10 $$S_K(X) = \left\{ {\begin{array}{*{20}{l}} {{\mathrm{sin}}(X)} \hfill & {{\mathrm{\Omega }}_k < 0}, \hfill \\ X \hfill & {{\mathrm{\Omega }}_k = 0}, \hfill \\ {{\mathrm{sinh}}(X)} \hfill & {{\mathrm{\Omega }}_k > 0,} \hfill \end{array}} \right.$$

and *E*(*z*) ≡ *H*(*z*)/*H*_0_. In addition, we denote *d*(*z*) ≡ *d*(0, *z*), *d*_l_ ≡ *d*(0, *z*_l_), *d*_s_ ≡ *d*(0, *z*_s_), and *d*_ls_ ≡ *d*(*z*_l_, *z*_s_), respectively. If the function of redshift *z* with respect to cosmic time *t* is single-valued and *d*′(*z*) > 0, these three distances in the FLRW scenario satisfy a simple sum rule^24^

11 $$d_{\mathrm{ls}} = d_{\mathrm{s}}\sqrt {1 + {\mathrm{\Omega }}_kd_{\mathrm{l}}^2} - d_{\mathrm{l}}\sqrt {1 + {\mathrm{\Omega }}_kd_{\mathrm{s}}^2} .$$

Apparently, one has *d*_s_ > *d*_l_ + *d*_ls_, *d*_s_ = *d*_l_ + *d*_ls_ and *d*_s_ < *d*_l_ + *d*_ls_ for Ω_*k*_ > 0, Ω_*k*_ = 0 and Ω_*k*_ < 0, respectively. Furthermore, in order to compare this sum rule with observations, we can rewrite Eq. (11) as

12 $$\frac{{d_{\mathrm{ls}}}}{{d_{\mathrm{s}}}} = \sqrt {1 + {\mathrm{\Omega }}_kd_{\mathrm{l}}^2} - \frac{{d_{\mathrm{l}}}}{{d_{\mathrm{s}}}}\sqrt {1 + {\mathrm{\Omega }}_kd_{\mathrm{s}}^2} .$$

Recently, the sum rule in this form was proposed to model-independent test, the FLRW metric, by comparing the distance ratios *d*_ls_/*d*_s_ measured from strongly lensed quasar systems with distances estimated from SNe Ia observations^25^. More recently, we upgraded the distance sum rule and rewrote it as^26^

13 $$\frac{{d_{\mathrm{ls}}}}{{d_{\mathrm{l}}d_{\mathrm{s}}}} = T(z_{\mathrm{l}}) - T(z_{\mathrm{s}}),$$

where

14 $$T(z) = \frac{1}{{d(z)}}\sqrt {1 + {\mathrm{\Omega }}_kd^2(z)} ,$$

to test the FLRW metric and estimate the cosmic curvature with time-delay distance measurements.

### Statistical analysis

In order to estimate constraining power from 10 lensed FRB systems, we propagate the relative uncertainties of time delay (*δ*Δ*τ* = 0), Fermat potential difference (*δ*ΔΦ = 0.8%), and LOS contamination (*δκ*_ext_ = 2%) to the relative uncertainty of *D*_Δ*t*_, and then to the (relative) uncertainties of cosmological parameters: $(\delta {\mathrm{\Delta }}t,\delta {\mathrm{\Delta \Phi }},\delta \kappa _{{\mathrm{ext}}})\sim \delta D_{{\mathrm{\Delta t}}}\sim (\delta H_{\mathrm{0}},\sigma _{{\mathrm{\Omega }}_k})$. Then, we performed Markov Chain Monte Carlo (MCMC) minimization of the following *χ*^2^ objective function:

15 $$\chi ^2 = \mathop {\sum}\limits_{i = 1}^{10} \left( {D_{{\mathrm{\Delta}t},i}^{{\mathrm{th}}}(z_{{\mathrm{d}},i},z_{{\mathrm{s}},i};{\mathbf{p}}) - D_{{\mathrm{\Delta}t},i}^{{\mathrm{sim}}}} \right)^2/\sigma _{D_{{\mathrm{\Delta}t},i}}^2,$$

where $D_{{\mathrm{\Delta}t}}^{{\mathrm{th}}}$ is the theoretical time-delay distance in the assumed cosmological model or from the combination of distance sum rule and SNe Ia observations, while $D_{{\mathrm{\Delta}t}}^{{\mathrm{sim}}}$ is the corresponding simulated distance with its uncertainty being $\sigma _{D_{{\mathrm{\Delta}t},i}} = \delta D_{{\mathrm{\Delta}t},i}D_{{\mathrm{\Delta}t},i}$. **p** represents cosmological parameters (*H*_0_, Ω_*k*_) to be constrained and they are sampled in ranges *H*_0_ ∈ [0, 150], Ω_*k*_ ∈ [−1, 1].

We perform 10,000 simulations each containing 10 lensed FRB systems. For each dataset, we carry out the above-mentioned MCMC minimization to obtain the best-fit value of corresponding parameters. Then, we plot the probability distributions of the best-fit *H*_0_ and Ω_*k*_ in Figs. 1, 2, respectively.

## Data Availability

The data that support the findings of this study are available from the corresponding author upon request.
